# Validating GAN-BioBERT: A Methodology for Assessing Reporting Trends in Clinical Trials

**DOI:** 10.3389/fdgth.2022.878369

**Published:** 2022-05-24

**Authors:** Joshua J. Myszewski, Emily Klossowski, Patrick Meyer, Kristin Bevil, Lisa Klesius, Kristopher M. Schroeder

**Affiliations:** ^1^School of Medicine and Public Health, University of Wisconsin, Madison, WI, United States; ^2^University of Wisconsin-Milwaukee, Milwaukee, WI, United States; ^3^Department of Anesthesiology, School of Medicine and Public Health, University of Wisconsin, Madison, WI, United States

**Keywords:** sentiment analysis, publication bias, natural language processing, clinical trial, meta-analyses

## Abstract

**Background:**

The aim of this study was to validate a three-class sentiment classification model for clinical trial abstracts combining adversarial learning and the BioBERT language processing model as a tool to assess trends in biomedical literature in a clearly reproducible manner. We then assessed the model's performance for this application and compared it to previous models used for this task.

**Methods:**

Using 108 expert-annotated clinical trial abstracts and 2,000 unlabeled abstracts this study develops a three-class sentiment classification algorithm for clinical trial abstracts. The model uses a semi-supervised model based on the Bidirectional Encoder Representation from Transformers (BERT) model, a much more advanced and accurate method compared to previously used models based upon traditional machine learning methods. The prediction performance was compared to those previous studies.

**Results:**

The algorithm was found to have a classification accuracy of 91.3%, with a macro F1-Score of 0.92, significantly outperforming previous studies used to classify sentiment in clinical trial literature, while also making the sentiment classification finer grained with greater reproducibility.

**Conclusion:**

We demonstrate an easily applied sentiment classification model for clinical trial abstracts that significantly outperforms previous models with greater reproducibility and applicability to large-scale study of reporting trends.

## Introduction

Publication bias is a systematic phenomenon of under or overreporting of research findings dependent on the direction of the results found (1). As a result of this phenomenon, systematic reviews of clinical guidelines may reach incorrect conclusions (2), and subsequently lead to harm to patients caused by treatments that have an otherwise poor evidence base. Despite this potential for harm and its widespread presence within clinical literature (1), there have been limited efforts to develop and utilize methods to characterize publication bias, particularly on a systematic scale. In 2016 Hedin et al. found that only 55 percent of meta-analysis in anesthesiology journals discussed publication bias, and only 43 percent actually used tools to assess the phenomenon (3). Furthermore, the methods currently used for assessing publication bias, such as funnel-plot based methods and selection models (4, 5), are criticized as unintuitive to interpret within the literature's context (4). These methods also focus on the quantitative findings expressed in the studies in question in the form of effect sizes and *p*-values and are therefore limited to those studies that express these types of findings.

The current gold standard for systematic assessment of the qualitative interpretation of the findings has been rating systems performed by human raters. However, this method of assessment is time and resource intensive and has inherently poor reproducibility due to variability between the raters used (6–8). Fortunately, this is changing with the development of sentiment analysis and natural language processing as a toolset capable of understanding the qualitative statements made in a body of text with consistency and accuracy, creating a promising avenue to address these shortcomings.

In recent years, several studies have explored the assessment of citation sentiment analysis in academic literature (9–12) with the goal of examining the sentiment toward papers cited in the body of another article as an assessment of article impact. Sentiment analysis has also been applied to the analysis of clinical notes in the electronic health record with the goal of prognostication (13, 14). However, attempts to use sentiment analysis to characterize the qualitative findings authors express toward their own clinical publication's findings have been minimal, with only two studies being published at the time of writing of this manuscript (15, 16). Importantly, the model accuracy in both cited studies were limited by the technology available at the time and the availability of labeled abstract data for training of the algorithms developed. Similarly the algorithms classes being limited to the two class tasks of positive/neutral (15) or positive/not positive (16) respectively, limited their practical use. The methods used in these studies did not take advantage of newer natural language processing architectures such as the context-sensitive Bidirectional Encoder Representations from Transformers (BERT) model (17), or similar newer models for the analyses built for biomedical text (18), instead opting for the use of a support vector machine (15) and a sequential neural network (16), respectively.

With the limitations of these previous studies in mind, this study's goal was to develop and validate a sentiment analysis model for clinical trial abstracts that can be practically applied to large-scale assessment of clinical literature using the more modern GAN-BERT architecture (19). This model is a semi-supervised approach to fine tuning a BERT model, taking advantage of both the decreased sample size required due to a semi-supervised approach, and the increased accuracy that the BERT architecture has become known for (17). Developing a tool with reproducible results for systematic large-scale assessment of reporting trends in clinical literature in this manner is a large step forward in actually addressing the issue of biased reporting in clinical literature and it's subsequent harm to patients.

## Methods

### Creation of the Labeled and Unlabeled Training Sets

There are no publicly available annotated datasets specific to sentiment analysis of clinical trials, so for the purposes of this study an appropriate annotated dataset had to be created. Given that the best raters for the sentiment rating of clinical trials are trained clinician experts, creation of a fully annotated dataset for this study was determined to be particularly resource intensive, a problem inherent to clinically related natural language processing (NLP) tasks (20). As such, this study elected to use a semi-supervised approach, combining expert-annotated clinical trial abstracts and large amounts of unlabeled data to minimize the resources required to create the final algorithm.

### Data Gathering and Annotation

All abstracts gathered for this study were from the National Library of Medicine's (NLM) PubMed database, filtered specifically to publications classified as clinical trials. The collection of these abstracts was automated using the NCBI's Entrez search and retrieval system with a data mining tool built by the authors using the BioPython toolkit (21). This tool can gather all MEDLINE data that is reported for a particular PubMed query, and is able to search in a specific medical field by cross referencing journal ID numbers with a NLM catalog query.

For the creation of the labeled dataset, 12 abstracts each from clinical trials in the fields of Obstetrics & Gynecology, Orthopedics, Pediatrics, Anesthesiology, General Surgery, Internal Medicine, Thoracic Surgery, Critical Care, and Cardiology were randomly selected, for a total of 108 labeled abstracts. These abstracts were then stripped of all information other than the abstract text and provided to a panel of three clinicians with a range of 9–19 years of experience to independently label the abstracts as having positive, negative, or neutral sentiment, with examples shown in Table 1. The ground truth class of these abstracts was then defined by the most common rating assigned to the abstract by a panel of three clinicians, plus a fourth to review abstracts when there was not a majority decision between the other three. The smallest and largest class of the labeled data was then oversampled or undersampled to equal the number of samples from the median class to create an algorithm with a maximally balanced accuracy between classes (22). The unlabeled dataset was a collection of 2,000 clinical trial abstracts selected from PubMed in the same manner described above, excluding those used in the labeled dataset. The unlabeled data is then given a label of UNK UNK so that when it is used to train the classification algorithm the label is appropriately masked.

**Table 1:** Examples of positive, negative, and neutral text in abstracts.

**Example**	**Classification**
This study showed promising results regarding treatment A	Positive
This study showed no significant difference between Treatment A and Treatment B	Negative
This study showed that treatment A is inappropriate for common use	Neutral

### Data Preprocessing

The conclusion sentences of the labeled and unlabeled abstracts to be used for training and validation were then extracted. This was done as it was found in previous study that using solely the concluding sentences led to an increase in classification accuracy (15, 16). Using the Natural Language Tool Kit (NLTK) Python toolkit (23), concluding sentences were identified as those following the conclusion heading for structured abstracts. For unstructured abstracts, the conclusion sentences were determined to be the last n sentences of an abstract based on the number of sentences in the abstract using equation 1 below, where St is the total number of sentences.


(1) $$n=ceil\left(St^{*}0.125\right)$$

The relative value of 0.125 was determined empirically in a previous study based on the analysis of 2,000 structured abstracts (15).

Tokenization: Following extraction of the conclusion sentences, all sentences were tokenized using the BERT tokenizer available as part of the HuggingFace Transformers toolkit (24), which tokenizes each word. The BERT tokenizer begins by tagging the first token of each sentence with the token [CLS], then converting each token to its corresponding ID that is defined in the pre-trained BERT model. The end of each sentence is then padded with the tag [PAD] to a fixed sentence length, as the BERT model requires a fixed length sentence as an input (17).

### GAN-BioBERT Workflow

**Figure 1:**
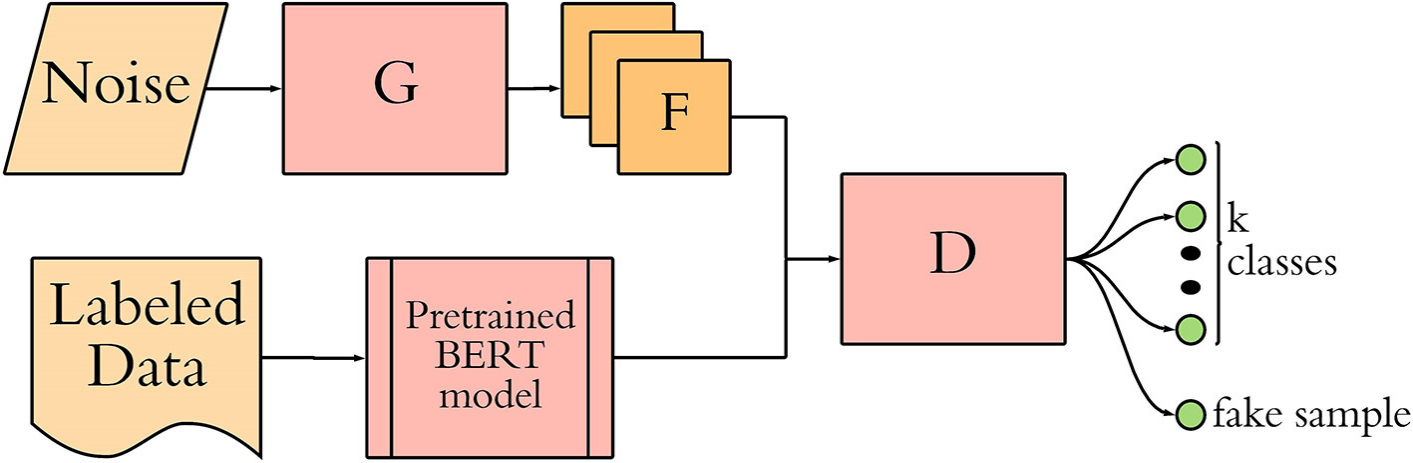
A visual representation of the GAN-BERT algorithm as described by the original developers where G, Generator D; Discriminator; F, Fake Sample (19).

Generally, the GAN-BERT architecture consists of a generator function G based on the Semi-Supervised generalized adversarial network (GAN) architecture that generates fake samples F using a noise vector as input (25), the pre-trained BERT model, which is given the labeled data, and a discriminator function D that is a BERT-based k-class classifier that is fine-tuned to the classification task (19). This workflow is shown graphically in Figure 1, with further discussion of each element to follow. The GAN-BioBERT architecture as it is written by its original creators uses HuggingFace transformers as the basis for it's creation in python, which is also what was used in this study (19).

### BERT Architecture

Before discussing the details of the algorithm used in this study it is key to first discuss the general BERT architecture. Bidirectional Encoder Representations from Transformers or BERT model is a method for language processing first described in 2018 by Devlin et al. that achieved state of the art performance on a variety of natural language processing tasks and has since become a heavily used tool in natural language processing research (17). BERT functions using 2 sequential workflows, a semi-supervised language modeling task that develops a general language model, then a supervised learning step specific to the language processing task the model is being applied to such as text classification. For developing the pre-trained language model BERT is provided with a very large corpus from a particular domain, such as publications in PubMed (18), documents from a particular language (26), or English Wikipedia and BooksCorpus as in the original BERT model (17). BERT then develops a complete language model from the provided corpus using both masked language modeling, which determine the meaning of individual words within the sentence's context, and next sentence prediction, which works to understand the relationship between sentences. The result of this process is a trained context-sensitive general language model for the specific domain being studied that can then be disseminated for a wide variety of applications. The pretrained language model from the semi-supervised stage of BERT is then fine-tuned for a specific language task by providing task-specific inputs and outputs and then adjusting the parameters of the model accordingly to create the complete task-specific algorithm (17).

### BERT Pretrained Model Selection

Given the important role of the pretrained model in the BERT architecture, and the relative complexity of biomedical literature, general language models are likely to encounter lower accuracy when applied to a biomedical application such as the one in this study due to a change in the word distributions between general and biomedical corpora (18). As such, in this study the pretrained BioBERT model was used as the general language model to be fine-tuned for sentiment classification (18). BioBERT is a 2020 pretrained BERT model by Lee et al. that is specific to the biomedical domain that was trained on PubMed abstracts and PubMed Central full-text articles, as well as English Wikipedia and BooksCorpus as was done in the original BERT model (17, 18). As a result of this domain specific training, BioBERT has shown improved performance on a variety of biomedical NLP tasks when compared to the standard BERT models (18).

### GAN-BERT

While BERT and its derivatives have been able to achieve state of the art performance on a variety of tasks, one major limitation of the model is that fully trained models typically require thousands of annotated examples to achieve these results (19). Significant drops in performance were observed when <200 annotated examples are used (19). In order to address this limitation, Croce, Castellucci, and Basili developed the GAN-BERT model in 2020 as a semi-supervised approach to fine tuning BERT models that achieves performance competitive with fully supervised settings (19). Specifically, GAN-BERT expands upon the BERT architecture by the introduction of a Semi-Supervised Generative Adversarial Network (SS-GAN) to the finetuning step of the BERT architecture (25). In a SS-GAN, a “generator” is trained to produce samples resembling the data distribution of the training data i.e., the labeled abstracts in this study. This process is dependent on a “discriminator,” a BERT-based classifier in the case of this study, which in an SS-GAN is trained to classify the data into their true classes, in addition to identifying whether the sample was created by the generator or not. When trained in this manner, the labeled abstract data was used to train the discriminator, while both the unlabeled abstracts and the generated data is used to improve the model's inner representations of the classes, which subsequently increases the model's generalizability to new data (19). As a result of this approach the minimum number of annotated samples to train a BERT model is reduced from thousands, to a few dozen (19). Because of this effect, this study uses GAN-BERT to minimize the resource intensive process of creating an expert-annotated corpus of clinical trial abstracts. A detailed mathematical description of this algorithm and it's processes, including the determination of it's loss functions, can be found elsewhere (19).

In summary, in this study GAN-BioBERT takes the BERT architecture pretrained on biomedical text using BioBERT (18) and fine-tunes it for sentiment classification of clinical trial abstracts in a semi-supervised manner by using adversarial learning in the form of an SS-GAN architecture known as GAN-BERT (19, 25). The training data used consisted of a set of clinical trial abstracts annotated by three expert raters as positive, negative, or neutral, where the least common class was upsampled and the most common class was downsampled to create a balanced training set, as well as 2000 (121,856 tokens) unlabeled clinical trial abstracts. The validation accuracy and F1-scores of the resulting algorithm were then determined and compared to both previous attempts at applying sentiment analysis to the findings in clinical trial abstracts, as well as the performance of a fourth expert rater on the same labeled data used to train and validate the algorithm, and the original GAN-BERT algorithm without BioBERT (i.e., with the standard BERT pretrained model).

## Results

Of the 108 abstracts (4,674 tokens) labeled by the expert raters, 26 were classified as positive, 69 were classified as neutral, and 13 were classified as negative by the raters. As such, the negative samples were up-sampled, and the neutral samples were down-sampled so that each class contained 26 examples, for a final labeled dataset of 78 abstracts for training purposes. In order to have a test set with a distribution similar to what is present in application of the algorithm, 23 of the samples were held out as the test set for determining the performance of the algorithm prior to balancing of the training dataset.

**Figure 2:**
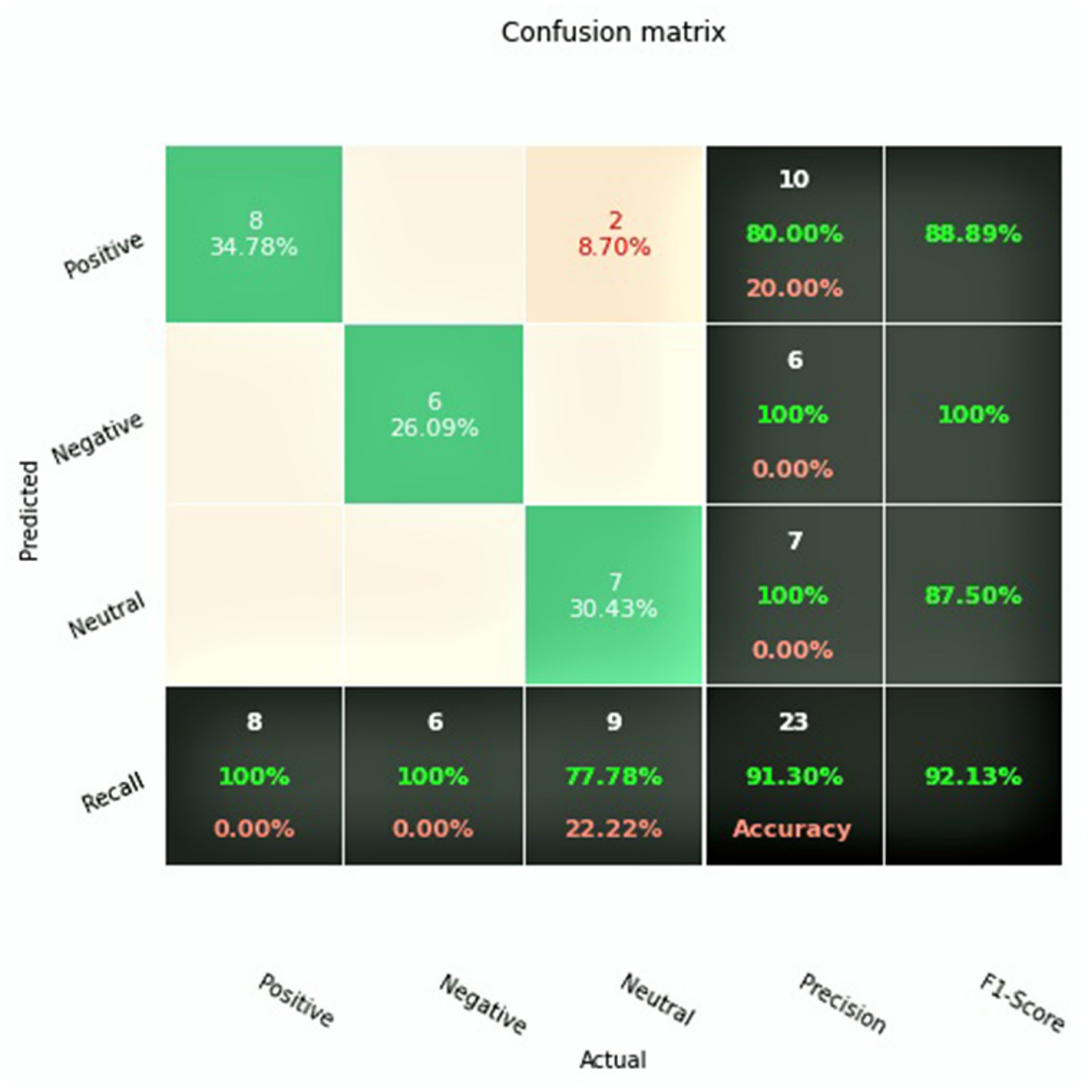
Confusion matrix for GAN-BioBERT.

After completion of training, the final GAN-BioBERT algorithm was found to have an accuracy of 91.3%, and a macro F1-Score of 0.92. The training of the algorithm took 45 min using the Google Colaboratory Environment using 35 GB of RAM with TPU hardware acceleration. The confusion matrix associated with these results is shown in Figure 2.

GAN-BERT using the base uncased BERT pretrained model was found to have an accuracy of 82.6% and a macro F1-score of 0.824. These results, alongside the results of the two previous studies investigating sentiment analysis of clinical trial abstracts, are summarized in Table 2.

**Table 2:** Performance metric results for both this study and previous studies.

**Study**	**Classification Method**	**Classification Type (# of classes)**	**Accuracy**	**F1-Score**
Fischer and Steiger (16)	Word Frequency + Sequential Neural Network	Positive, Not Positive (2)	73%	N/A
Zlabinger et al. (15)	Uni-gram Features + Support Vector Machine (SVM)	Positive, Neutral (2)	76%	0.72
This study, 2021	GAN-BERT	Positive, Negative, Neutral (3)	82.6%	0.824
This study, 2021	GAN-BioBERT	Positive, Negative, Neutral (3)	91.3%	0.92

## Discussion

From a technical perspective, these results show that GAN-BioBERT is a significant step forward for assessing the sentiment in clinical trial literature, with an 8.7% improvement in performance over GAN-BERT for the same classification task. This improvement in domain specific classification performance with creation of a domain specific algorithm is reasonable to expect but is important to assure the algorithms viability in an application where highly specific and technical language is commonplace. Beyond this, a reliable, rapid assessment method for clinical literature is a large step forward in the process of assessing trends in clinical literature as traditionally the assessment of clinical literature has been performed manually which creates significant resource and time restrictions on larger literature reviews. This also provides a reliable method of assessing potential biases in the literature by being able to operationalize some amount of subjective assessment of the literature using artificial intelligence.

When technically compared to previous studies' attempts at classifying sentiment in clinical trial abstracts (15, 16), this improvement is even more significant as there is an absolute accuracy improvement of 15.3%, while also expanding the classification task to the three classes positive, negative, and neutral, as opposed to the two-class positive/not positive (16), or positive/neutral (15). This significant improvement in accuracy and expansion of the number of classifiers make GAN-BioBERT much more suitable for large-scale assessment of the sentiment in clinical trial literature with improved accuracy and data resolution. With the already high classification accuracy of the algorithm in mind, further development of this algorithm technically may include the introduction of finer-grained sentiment classification, as well as the use of a larger set of labeled training data with more expert raters contributing to improve inference performance given the subjectivity of the task.

## Conclusion

This study presents GAN-BioBERT, a sentiment analysis classifier for the assessment of the sentiment expressed in clinical trial abstracts. GAN-BioBERT was shown to significantly outperform previous attempts to classify sentiment in clinical trial abstracts using sentiment analysis with regards to accuracy and number of sentiment classes. Considering this high multi-class accuracy, and the reproducible results GAN-BioBERT generates, this study posits GAN-BioBERT as a viable tool for large-scale assessment of the findings expressed in clinical trial literature in a way that was not previously possible, making a needed step forward in the methods used to address the important and patient-impacting issue of reporting bias in clinical literature. By using a tool such as GAN-BioBERT the large-scale assessment of qualitative reporting trends in clinical trial literature becomes significantly more feasible with more reproducible findings when compared to the past practice of manual assessment of reporting bias.

## Data Availability Statement

The model generated for this study can be found in the Zenodo repository at: https://doi.org/10.5281/zenodo.5699018.

## Author Contributions

JM, EK, and KS were responsible for conception and design. KS was responsible for administrative support. PM, KB, LK, and KS were responsible for provision of study materials. JM and EK were responsible for collection and assembly of data. JM was responsible for data analysis and interpretation. All authors were responsible for manuscript writing as well as final approval of the manuscript.

## Funding

This study was funded by the University of Wisconsin School of Medicine and Public Health's Shapiro Summer Research Program.

## Conflict of Interest

The authors declare that the research was conducted in the absence of any commercial or financial relationships that could be construed as a potential conflict of interest.

## Publisher's Note

All claims expressed in this article are solely those of the authors and do not necessarily represent those of their affiliated organizations, or those of the publisher, the editors and the reviewers. Any product that may be evaluated in this article, or claim that may be made by its manufacturer, is not guaranteed or endorsed by the publisher.
